# The Relationship between Sleep Bruxism Intensity and Renalase Concentration—An Enzyme Involved in Hypertension Development

**DOI:** 10.3390/jcm9010016

**Published:** 2019-12-19

**Authors:** Helena Martynowicz, Mieszko Wieckiewicz, Rafal Poreba, Anna Wojakowska, Joanna Smardz, Lidia Januszewska, Iwona Markiewicz-Gorka, Grzegorz Mazur, Krystyna Pawlas, Pawel Gac

**Affiliations:** 1 Department of Internal Medicine, Occupational Diseases, Hypertension and Clinical Oncology, Wroclaw Medical University, 50-556 Wroclaw, Poland; helenamar@poczta.onet.pl (H.M.); sogood@poczta.onet.pl (R.P.); ania.wojakowska@wp.pl (A.W.); grzegorzmaz@yahoo.com (G.M.); 2 Department of Experimental Dentistry, Wroclaw Medical University, 50-425 Wroclaw, Poland; joannasmardz1@gmail.com; 3 Department of Hygiene, Wroclaw Medical University, 50-345 Wroclaw, Poland; lidia.januszewska@umed.wroc.pl (L.J.); iwona.markiewicz-gorka@umed.wroc.pl (I.M.-G.); krystyna.pawlas@umed.wroc.pl (K.P.); pawelgac@interia.pl (P.G.)

**Keywords:** renalase, sleep, sleep bruxism, hypertension

## Abstract

Background and objectives: Renalase, a novel amine oxidase, is involved in the development of hypertension. Sleep bruxism (SB) is a sleep-related behavior characterized by rhythmic or non-rhythmic activity of the masticatory muscles, which leads to the mechanical wear of teeth, pain in the masticatory muscles, and disturbed sleep. Recent studies indicate that SB plays a role in increased blood pressure. Therefore, this study aimed to determine the relationship between sleep bruxism intensity and renalase concentration, which may help in the future to elucidate the pathogenesis of hypertension and other cardiovascular disorders. Material and methods: SB was evaluated in 87 adult patients using single-night diagnostic polysomnography with video and audio recordings, and the episodes of bruxism were scored according to the standards of the American Academy of Sleep Medicine. The levels of serum renalase were measured in the patients using enzyme-linked immunosorbent assay kits. Results: SB (Bruxism Episode Index (BEI) ≥2) was diagnosed in 54% (n = 47) of the studied population, and the mean concentration of renalase was found to be decreased in the hypertensive group compared with the normotensive group (133.33 ± 160.71 vs 219.23 ± 220.58, *p* = 0.047). In addition, a linear negative correlation was observed between the renalase concentration and the body mass index (BMI) in the SB group (r = 0.38, *p* < 0.05) but not in controls. Thus, higher BEI and higher BMI were identified as factors independently associated with the lower concentration of renalase, but only in the group of patients which had a blood renalase concentration of >212.5 ng/mL. Conclusion: There exists an association between renalase concentration and SB intensity, and further studies are needed to clarify the role of renalase in the pathogenesis of hypertension and other cardiovascular disorders.

## 1. Introduction

Renalase, a novel amine oxidase, plays a crucial role in the pathogenesis of human diseases. This enzyme degrades circulating catecholamines and can be detected in the kidneys, heart, skeletal muscles, small intestine, brain, and peripheral nervous system. The best substrate of renalase is dopamine, followed by epinephrine, and then norepinephrine [1]. Catecholamines are involved in the activation of the sympathetic nervous system and also in the development of arterial hypertension, and thus, renalase indirectly regulates blood pressure by degrading these substrates. It was reported that a decrease in the gene expression of renalase by about 40% is followed by an increase in blood pressure by 13 mmHg [2]. In addition, renalase is believed to be involved in the regulation of the intrarenal dopaminergic system [3]. Consequently, it was proposed that renalase regulates blood pressure either by downregulating the activity of the sympathetic nervous system or by degrading renal dopamine [4].

Sleep bruxism (SB) is a sleep-related behavior, characterized by rhythmic or non-rhythmic activity of the masticatory muscles, which commonly leads to the mechanical wear of teeth, pain in the jaw muscles, and disturbed sleep. The latest “International consensus on the assessment of bruxism” has defined SB as a “masticatory muscle activity during sleep that is characterized as rhythmic (phasic) or non-rhythmic (tonic) and is not a movement disorder or a sleep disorder in otherwise healthy individuals” [5]. A considerable number of risk factors have been identified for SB, which include caffeine, smoking, stress, alcohol, anxiety, age, drugs [6,7], and obstructive sleep apnea [8]. Recently, our research group found that diabetes [8], body mass index (BMI), and hypertension [9] act as independent risk factors for SB [9]. In a study by Nashed, SB was shown to be associated with an increase in blood pressure during the rhythmic activity of the masticatory muscles [10].

However, the relationship between renalase and sleep bruxism remains unclear. Therefore, we aimed to assess the relationship between sleep bruxism intensity and renalase concentration, which may help in the future to elucidate the pathogenesis of hypertension and other cardiovascular disorders.

## 2. Material and Methods

We enrolled 87 adult patients hospitalized due to suspected SB in the Sleep Laboratory in the Department of Internal Medicine, Occupational Diseases, Hypertension and Clinical Oncology at Wroclaw Medical University, Poland. The inclusion criteria considered for the study were as follows: age over 18 years, absence of contraindications for polysomnography (PSG) examination, and willingness to participate in the study. The exclusion criteria considered were as follows: presence of severe systemic diseases, presence of secondary bruxism induced by systemic diseases and/or drugs, use of medicines that can significantly affect the function of the nervous and muscular systems, presence of severe mental illness or significant mental disorders, and presence of active malignancy.

The single-night diagnostic PSG examination was conducted using Nox-A1 (Nox Medical, Iceland) in the Sleep Laboratory at Wroclaw Medical University. Polysomnograms were assessed in 30-s epochs according to the standard criteria for sleep scoring proposed by the American Academy of Sleep Medicine (AASM) [11]. The following outcome variables of PSG were assessed: sleep latency (min), total sleep time (min), sleep efficiency (%), and the percentages of N1, N2, N3, and rapid eye movement (REM) sleep. SB was assessed by electromyography (EMG) of the bilateral masseter muscles and by evaluating video and audio recordings. Bruxism episodes were scored according to the AASM standards and categorized as follows: phasic, tonic, and mixed. To confirm the diagnosis of SB, the EMG activity should be at least twice the amplitude of the background activity, and the EMG bursts should not be separated by >3 s to be considered as part of the same episode. A constant burst episode sustained over 2 s in the EMG recording of the masseter muscles was categorized as tonic, an episode including three or more bursts sustained over 2 s was categorized as phasic, and an episode showing the characteristics of both tonic and phasic episodes was categorized as mixed [12]. The Bruxism Episode Index (BEI) measures the number of bruxism episodes per hour of sleep (<2: irrelevant SB; 2–4: mild/moderate SB; >4: severe SB) [13].

Venous blood samples were drawn from the participants the morning after an overnight PSG examination. The samples were stored at 20 °C until they were used for analysis. The levels of renalase in serum were measured using a commercially available enzyme-linked immunosorbent assay kit (Bioassay Technology Laboratory, Shanghai, China), according to the manufacturer’s instructions. The E3109Hu kit was used to carry out sandwich enzyme immunoassays for the in vitro quantitative measurement of human renalase (also known as RNLS) in serum, plasma, cell culture supernates, cell lysates, and tissue homogenates. Renalase concentration is expressed in ng/mL, and the standard range of detection is 1–400 ng/mL. The sensitivity of the kit is 0.52 ng/mL, and the coefficient of intra- and inter-assay variation is <8% and <10%, respectively.

Statistical analyses were conducted using Dell Statistica 13 software (Dell Inc., Round Rock, TX, USA). The quantitative variables were calculated as arithmetic means and standard deviations, while the qualitative variables were determined as percentages. The distribution of quantitative variables was verified using the Shapiro–Wilk W-test, while a t-test or the Mann–Whitney U-test was used for the evaluation of the independent variables in comparative analyses. The relationships between the analyzed variables were determined by correlation and multivariate segmented linear regression analysis with breakpoints. Results with *p* < 0.05 were considered statistically significant.

This study was approved by the Ethical Committee of Wroclaw Medical University (ID KB-195/2017) and was conducted in accordance with the Declaration of Helsinki. All patients signed an informed consent form before participating in the study. Clinical Trial Registration: www.ClinicalTrials.gov; identifier NCT03083405.

## 3. Results

The mean age of participants was 47.40 ± 14.93 years. Women constituted 48% (n = 42) of the study population. The mean BMI of the participants was found to be 28.45 ± 5.69 kg/m^2^. Obesity (BMI ≥30) was diagnosed in 40% (n = 24), and overweight (25 ≤ BMI ≤ 29.99) in 35% (n = 36) of the participants. Diabetes and ischemic heart disease were diagnosed in 6.8% (n = 6) and 5.7% (n = 5) of the study population, respectively, and 29% (n = 26) of the participants were diagnosed as hypertensive.

SB (BEI ≥ 2) was diagnosed in 54% (n = 47) of the study population, of which 32% (n = 28) had severe SB (BEI ≥ 2). The mean BEI was calculated as 4.04 ± 4.5, while the mean phasic, tonic, and mixed BEIs were 2.04 ± 3.26, 1.09 ± 1.24, and 0.72 ± 1.24, respectively. The polysomnographic parameters of the studied group are presented in Table 1.

The mean concentration of renalase in the entire study group was 193.56 ± 207.41 ng/mL. No statistically significant difference was observed in renalase concentration between the SB group (BEI > 2) and controls (BEI < 2) (218 ± 221 vs. 163.88 ± 187.67, *p* > 0.05).

The mean renalase concentration was found to be decreased among the hypertensive group compared with the normotensive group, over the entire study group (133.33 ± 160.71 vs. 219.23 ± 220.58, *p* = 0.047).

**Figure 1:**
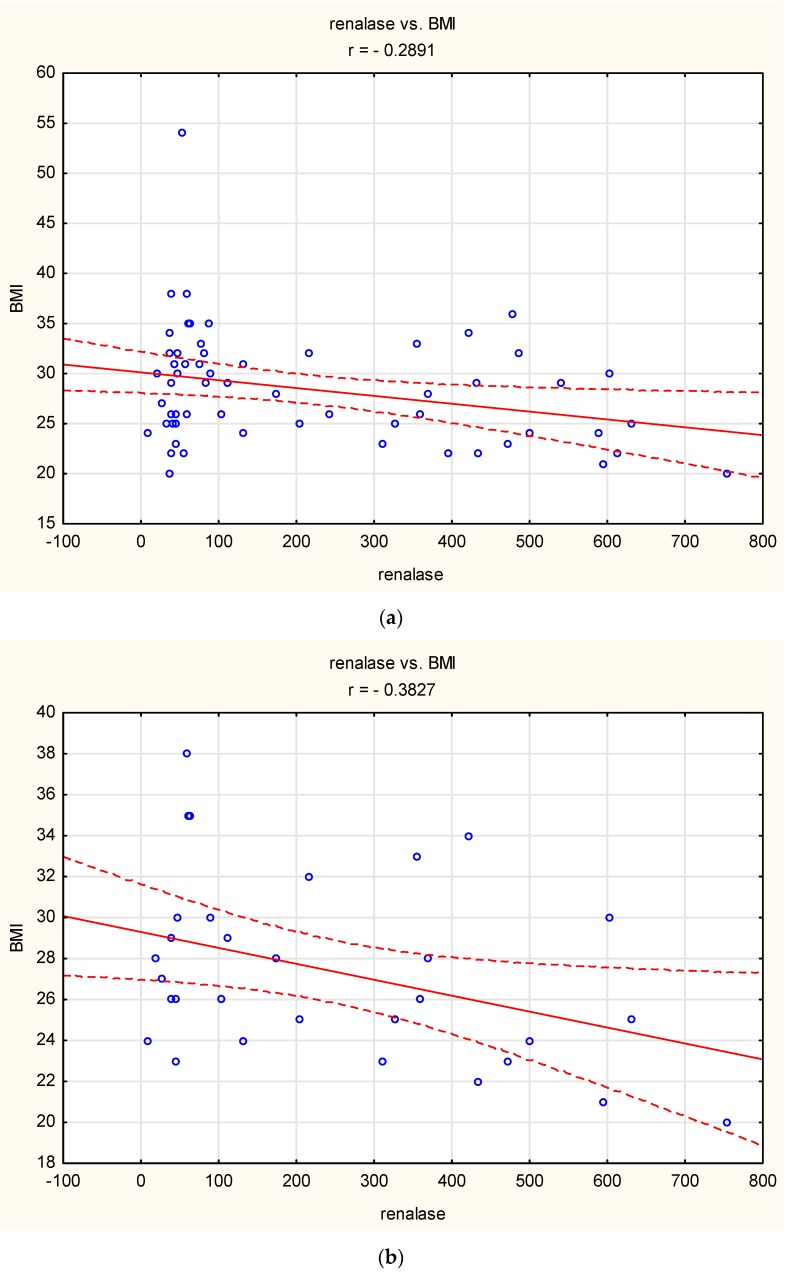
Correlation between Body Mass index (BMI) and renalase: (**a**) over the entire study group (*r* = −0.29, *p* < 0.05), (**b**) in the sleep bruxism (SB) group (*r* = −0.38, *p* < 0.05). Circle—single participant, solid line—trend line, dashed line—confidence interval 95%.

A linear negative correlation was observed between BMI and renalase concentration over the entire study group and in the SB group (r = 0.29, *p* < 0.05 and r = 0.38, *p* < 0.05, respectively) (Figure 1).

**Figure 2:**
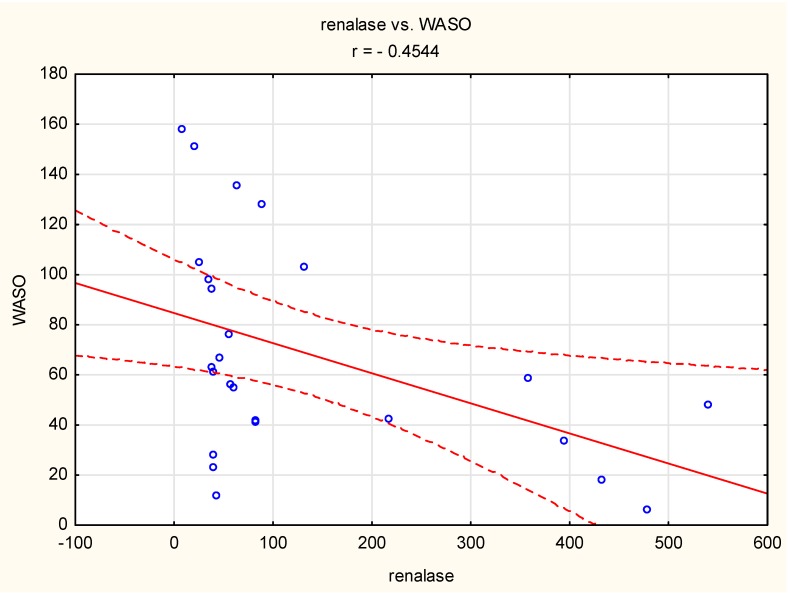
Correlation between “wake after sleep onset” (WASO) and renalase concentration (*r* = −0.45, *p* < 0.05) in the hypertensive group. Circle—single participant, solid line—trend line, dashed line—confidence interval 95%.

However, such a correlation was not observed in the normotensive group. In the case of the hypertensive group, “wake after sleep onset” (WASO) was negatively correlated with renalase concentration (r = 0.45, *p* < 0.05) (Figure 2).

**Figure 3:**
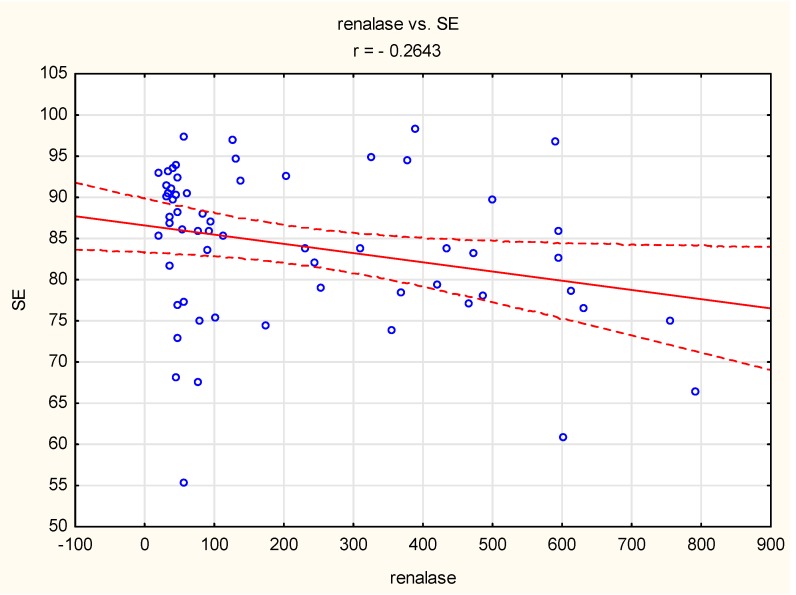
Correlation between sleep efficiency (SE) and renalase concentration (*r* = −0.26, *p* < 0.05) in the normotensive group. Circle—single participant, solid line—trend line, dashed line—confidence interval 95%.

A negative correlation was also observed between sleep efficiency and renalase concentration (r = 0.26, *p* < 0.05) in the normotensive group (Figure 3).

**Figure 4:**
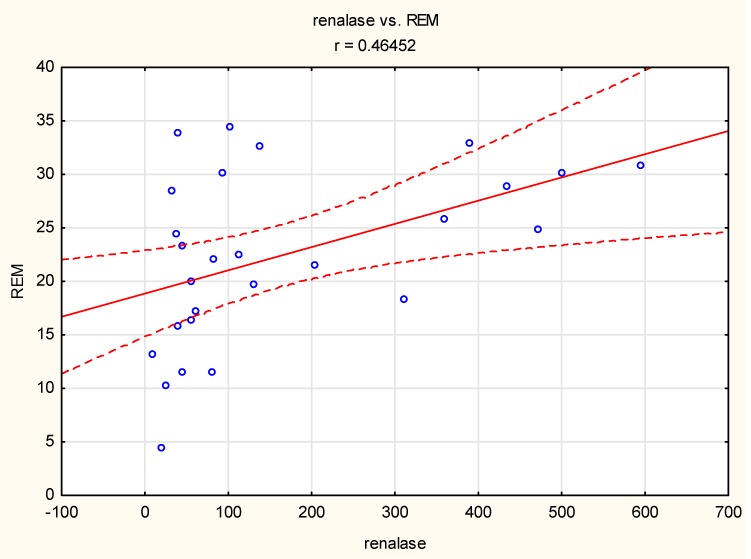
Correlation between the duration of rapid eye movement (REM) sleep and renalase concentration (r = 0.46, *p* < 0.05) in the group with severe bruxism. Circle—single participant, solid line—trend line, dashed line—confidence interval 95%.

In the group with severe bruxism, a positive linear correlation was observed between the duration of REM sleep and renalase concentration (r = 0.46, *p* < 0.05) (Figure 4).

**Figure 5:**
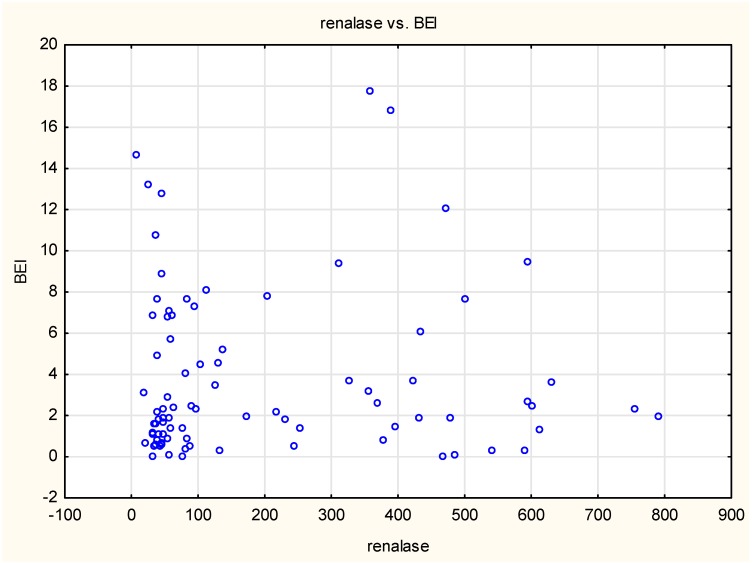
Relationship between Bruxism Episode Index (BEI) and renalase concentration over the entire study group.

The chart of BEI dependence on renalase concentration suggested a different type of relationship to a linear one (Figure 5).

Based on the multivariate segmented linear regression analysis with breakpoints performed for the entire study group, the following relationship model was obtained: renalase = 77.512-0.750 BEI-0.322 BMI for renalase < 212.514 ng/mL (*p* < 0.05) and 806.565-9.592 BEI-11.582 BMI for renalase > 212.514 ng/mL (*p* < 0.05).

The obtained model indicated that higher BEI and higher BMI were independently associated with lower blood concentration of renalase, but only in the group of patients with renalase concentration >212.514 ng/mL. At lower blood concentrations of renalase (<212.514 ng/mL), the relationship between BEI and renalase concentration was statistically insignificant. The estimation results for the model obtained in the multifactorial segmented regression analysis are presented in Table 2.

## 4. Discussion

The most important finding of this study was that renalase concentration was lesser in hypertensive patients compared to normotensive patients. It has previously been shown that renalase deficiency may be related to an increase in the levels of dopamine, epinephrine, and norepinephrine, and elevated blood pressure, leading to hypertension [1]. SB is commonly accompanied by excessive levels of catecholamines [14]. In addition, bruxism events are accompanied by a surge in blood pressure [10]. Thus, our results are in agreement with those of previous studies and might indicate the role of renalase in the development of hypertension.

A negative linear correlation between renalase concentration and BMI was observed only in the SB group (BEI > 2), whereas no such correlation was observed in the healthy controls (BEI < 2). A decreased concentration of catecholamines in obese subjects has also been reported in previous studies [15,16]. Thus, renalase might play a role in the reduction of catecholamines in sleep bruxers. The correlation observed in this study indicates that this pathomechanism might be more significant in subjects with lower BMI; however, we did not assess the concentration of catecholamines in this study.

We also found a correlation between renalase concentration and the duration of REM sleep in severe sleep bruxers (BEI > 4). REM sleep is marked by low-amplitude, high-frequency electroencephalographic rhythms, an active suppression of the activity of skeletal muscles, intermittent muscle twitches, an elevated arousal threshold, a brain activity that is more similar to wakefulness [17,18,19], and the activation of the autonomic nervous and respiratory systems [20,21,22,23]. REM sleep is also critically involved in memory consolidation [24,25,26,27]. Blood pressure is markedly altered in sleep—during non-REM sleep, blood pressure is decreased compared with during wakefulness, and is relatively stable [28,29], whereas during REM sleep, a puzzling response is observed and blood pressure is unstable and increases [29,30]. It was suggested that during REM sleep, diverse changes in sympathetic outflows might be needed to regulate blood pressure without the involvement of the heart [30]. Thus, the regulation of blood pressure during REM sleep is complex, and renalase activity might be one of the elements regulating blood pressure in sleep bruxers.

A negative correlation between renalase concentration and WASO was observed only in hypertensive patients, not in normotensive patients. WASO is defined as the time spent awake after sleep onset but before the final awakening [31]. This sleep parameter strongly influences sleep efficiency [31] and has a negative effect on sleep quality. The relationship between WASO and increased blood pressure has been described in previous studies. WASO has been found to be negatively correlated with a decrease in diastolic blood pressure [32]. This suggests that in hypertensive individuals, renalase might be involved in increases in blood pressure that are related to decreased sleep quality. However, we also observed a negative correlation between sleep efficiency and renalase concentration in the normotensive group, so the role of renalase in the regulation of blood pressure during sleep is complex and should be validated in larger studies.

Based on the multifactorial segmented regression analysis performed over the entire study group, we found that both a higher BEI and a higher BMI were independently associated with lower blood concentration of renalase, but only in the group of patients with a blood renalase concentration >212.5 ng /mL. This result might indicate the involvement of SB and increased BMI in the early stage of the development of hypertension. The role of these factors might decline with the decrease in renalase concentration, which is followed by an increase in blood pressure. To our knowledge, this is the first study to report the relationship between renalase concentration, hypertension, and SB, providing new insight into the pathophysiology of hypertension during sleep.

The main limitation of this study was the lack of recording of blood pressure using ambulatory blood pressure monitoring. Patients with obstructive sleep apnea were not excluded, which is a severe limitation of the study. Furthermore, the concentration of catecholamines was not determined.

## 5. Conclusions

The present study showed that there exists an association between renalase concentration and SB intensity. Renalase plays an important role in the pathomechanism of hypertension occurring during sleep, and further studies are needed to clarify its involvement in the pathogenesis of hypertension and other cardiovascular disorders.

## Figures and Tables

**Table 1:** Polysomnographic indexes of the studied group (n = 87).

Parameter	Mean ± SD	Minimum	Maximum
SE (%)	81.92 ± 10.08	52.40	98.30
SL (min)	22.32 ± 20.77	0.00	112.60
WASO (min)	49.77 ± 38.96	1.00	158.00
N1 (% of TST)	5.13 ± 4.48	0.30	21.20
N2 (% of TST)	45.94 ± 9.78	15.20	64.70
N3 (% of TST)	26.70 ± 10.68	2.60	54.90
REM (% of TST)	22.24 ± 7.68	4.10	38.40
BEI (n/hour)	4.03 ± 4.58	0.0	24.70
Phasic (n/hour)	2.04 ± 3.26	0.0	19.30
Tonic (n/hour)	1.09 ± 1.24	0.0	6.40
Mixed (n/hour)	0.72 ± 0.74	0.0	4.00
AHI (n/hour)	16.21 ± 17.18	0.0	77.00
ODI (n/hour)	16.31 ± 17.28	0.0	75.90
Mean SatO_2_ (%)	93.51 ± 2.42	83.30	97.30

SE—sleep efficiency, SL—sleep latency, WASO—wake after sleep onset, REM—rapid eye movement, BEI—Bruxism Episode Index, AHI—apnea-hypopnea index, ODI—oxygen desaturation index, SatO_2_ —oxygen saturation, TST—total sleep time.

**Table 2:** Results of estimation for the final model obtained in multivariate regression analysis.

Model for: Renalase (ng/mL)			
	Intercept	BEI (/Hour)	BMI (kg/m^2^)
Regression coefficient for renalase <212.514 ng/mL	77.512	−0.750	−0.322
*P*-value	<0.01	<0.05	<0.05
*P*-value of the model for renalase <212.514 ng/mL	<0.05		
Regression coefficient for renalase >212.514 ng/mL	806.565	−9.592	−11.582
*P*-value	<0.05	>0.05	<0.05
*P*-value of the model for renalase >212.514 ng/mL	<0.05		
*P*-value of the whole model	<0.05		
Determination coefficient (*R*^2^)	85.62%		
